# Corrosion behavior of zirconia in acidulated phosphate fluoride

**DOI:** 10.1590/1678-775720150435

**Published:** 2016

**Authors:** Anie Thomas, Sathyanarayanan Sridhar, Shant Aghyarian, Pilanda Watkins-curry, Julia Y. Chan, Alessandro Pozzi, Danieli C. Rodrigues

**Affiliations:** 1 - University of Texas at Dallas, Department of Biomedical Engineering, Richardson, USA.; 2 - University of Texas at Dallas, Department of Chemistry, Richardson, USA.; 3 - Polytechnic University of Marche, Department of Oral Surgery-Implant Dentistry, Ancona, Italy.

**Keywords:** Dental abutments, Dental implants, Dental crowns, Acidulated phosphate fluoride, Corrosion, Ceramics

## Abstract

**Objective:**

The corrosion behavior of zirconia in acidulated phosphate fluoride (APF) representing acidic environments and fluoride treatments was studied.

**Material and Methods:**

Zirconia rods were immersed in 1.23% and 0.123% APF solutions and maintained at 37°C for determined periods of time. Surfaces of all specimens were imaged using digital microscopy and scanning electron microscopy (SEM). Sample mass and dimensions were measured for mass loss determination. Samples were characterized by powder X-ray diffraction (XRD) to detect changes in crystallinity. A biosensor based on electrochemical impedance spectroscopy (EIS) was used to detect ion dissolution of material into the immersion media.

**Results:**

Digital microscopy revealed diminishing luster of the materials and SEM showed increased superficial corrosion of zirconia submerged in 1.23% APF. Although no structural change was found, the absorption of salts (sodium phosphate) onto the surface of the materials bathed in 0.123% APF was significant. EIS indicated a greater change of impedance for the immersion solutions with increasing bathing time.

**Conclusion:**

Immersion of zirconia in APF solutions showed deterioration limited to the surface, not extending to the bulk of the material. Inferences on zirconia performance in acidic oral environment can be elucidated from the study.

## INTRODUCTION

Zirconia and its alloys are gaining prevalence in the dental implant sector because of their aesthetic properties, biocompatibility, low plaque surface adhesion, high flexural strength, desirable osseointegration^10^, absence of mucosal discoloration^12^ and corrosion resistance^4^. The ongoing research for aesthetic, functionally stable and biocompatible materials has favored the use of all-ceramic reconstructions for fixed dental prostheses (FDPs) as alternatives to conventional porcelain-fused-to-metal (PFM) prostheses^11^. High-strength metal-oxide ceramics have been developed to overcome the mechanical drawbacks and high fracture rates of previous all-ceramic systems^23^. Ceramics are effectively replacing the metallic components of prosthetic restorations^16^. Alumina was the first bioceramic fabricated to serve as part of dental prosthetics; however, its inherent low bending strength and fracture toughness lead to failure due to the fractures. With higher fracture resistance and elastic modulus, zirconia became the material of choice succeeding alumina. Yttria-stabilized zirconium dioxide (Y-TZP) is more biocompatible than its metal alternatives, preventing soft tissue inflammation and discoloration^1^ ^,^ ^12^. White and shaded zirconia frameworks prevent bluish discoloration of peri-implant soft tissues and may be beneficial if soft tissue recession occurs in the long term. Thus, Y-TZP contributes to achieving healthy soft tissue integration of implant-supported restorations, thus improving long-term stability of the marginal bone^19^. Zirconia-based all-ceramics are currently used to fabricate copings, implant abutments, partial and complete arch frameworks on both natural teeth and implants, in both anterior and posterior oral cavity areas^13^ ^,^ ^17^ ^,^ ^19^ ^-^ ^21^. The success rates of these frameworks are comparable with conventional PFM FDPs, but it is important to note that these statistics are reports from three to five years of clinical use^15^.

In spite of the reported success, clinical reports of zirconia’s catastrophic failures do exist with significant biological and technical cost^3^. Generally, failures of ceramics are highly associated with fracture due to the inherent brittleness of the material^12^. Despite this, zirconia constitutes a superior fracture toughness stabilized by the transformation in the crystalline structure. However, there are certain environmental conditions that can lead to degradation of the material. One of the most important environmental degradation mechanisms associated with zirconia is known as Ageing (or) Low Temperature Degradation (LTD)^14^. The process of ageing takes place in two different scenarios: (i) stress corrosion: slow transformation of surface particles to the monoclinic phase over time; (ii) chemical degradation: a chemisorption of OH^-^ from water at the surface of zirconia grains to form Y(OH)_3_ and depletion of Yttrium. Transformation occurs in both scenarios and is often ensued by a nucleation and growth process. This phenomena can lead to a cascade of events such as crack growth on the surface which is termed Subcritical Crack Growth (SCG) or static fatigue^6^ ^,^ ^14^ ^,^ ^29^. These processes can trigger *in vivo* release of ceramic particles. Generally, ceramic particles are very inert and do not trigger adverse reactions, however there have been studies pointing to the ability of zirconia powder to cause inflammatory reactions due to the formation of zirconium hydroxide^16^. Furthermore, investigation of the presence of ceramic particles in aspirated synovial fluid is considered as a possible diagnostic tool for early detection of fracture in ceramic-on-ceramic (COC) hip implants^25^.

However, the formation of zirconium hydroxide has now been overcome with the sintering process^16^. Nevertheless, the main clinical concern reported in the literature regarding Y-TZP used as a framework material is a higher incidence of veneering porcelain chip-off fracture rates^10^ ^,^ ^11^, which has been reported to range from 15% to 54% over a 3- to 5-year period^11^ ^,^ ^12^, versus 2.9% to 8.8% ceramic fracture rates observed in conventional tooth- and implant-supported metal-ceramic restorations over 5 years^18^. Despite this mechanical complication, patient satisfaction, favorable soft tissue response and high aesthetic outcome were noted^18^. Several hypotheses concerning the causes of porcelain veneer chipping highlighted the importance of factors such as framework design, laboratory handling, baking procedures and ceramic mechanical properties^15^.

Hence, it is important to investigate and understand the effect of unfavorable acidic aqueous environment on the surface of newly incorporated materials such as zirconia. It has been also suggested that an aqueous environment with acidic conditions can accelerate the process of ageing with crack growth decreasing 20%-30% the prosthesis’ lifetime^4^. The oral environment is an aqueous electrochemical medium with high chances of pH fluctuations triggered by the presence of bacteria, ingestion of foods or presence of an inflammatory condition^26^. In addition to this, topical oral hygiene mouthwashes prescribed to patients contain highly acidic media due to sodium fluoride and hydrochloric acid^30^. Acidulated Phosphate Fluoride (APF) is one such common topical mouthwash used to combat dental caries. The effect of corrosive environments has been assessed for different dental ceramics where the low pH of the medium attacked the surface leading to coarser surfaces^24^. Although the influences of different pH values on zirconia have been evaluated, effects of clinical solutions, such as APF, on the surface of zirconia have not been investigated^28^.

Henceforth, this study focuses on the investigation of the surface characteristics of zirconia samples exposed to acidulated phosphate fluoride solutions (0.123% and 1.23%) for an extended time period of 11 d. This study is based on the hypothesis that an acidic aqueous medium in the oral environment can accelerate the process of ceramic ageing. This process can generate nucleation and propagation of cracks, in which fractures will result in dissolution of ceramic particles in the oral environment. Microscopic techniques were performed to understand the morphological features of the surface. X-ray diffraction (XRD) was employed to evaluate the crystal structure of the ceramic material because the change in the crystal structure is considered an indicator of material degradation. An electrochemical sensor was used to detect the presence of ceramic particles dissolution in the APF medium in which the samples were immersed. The overall goal of this study is to provide information on the performance of zirconia materials under simulated acidic oral environment.

## MATERIAL AND METHODS

### Material

A non-porous zirconia ceramic rod was obtained from a vendor (McMaster-Carr; Atlanta, Georgia, USA; 97% ZrO_2_, 3% trace elements) with diameter of approximately 0.15 cm and 30 cm in length. Acidulated phosphate fluoride (1.23%) (Pediagel, pH3.8, Indian Trail, North Carolina, USA) was used to prepare the immersion solutions.

### Preparation of the zirconia rods

Ten zirconia rod sections were cut to an approximate length of 2.54 cm each and the ends smoothed to touch and visually using 500-grit silica paper (Pace Technologies, 500-grit, Tucson, Arizona, USA). Before immersion, all the samples were immersed in acetone and sonicated for 5 min.

### Immersion setup and testing

The cleaned zirconia rods were divided into four groups involving different immersion conditions as follows:

Group 1: a zirconia rod not subjected to any immersion was used as a control.

Group 2: a zirconia rod immersed in deionized (DI) water.

Group 3: four zirconia rods immersed in 1.23% APF medium with each rod immersed in an isolated medium contained in separate glass vials.

Group 4: four zirconia rods immersed in 0.123% APF medium in a similar manner to group 3. 0.123% APF was prepared by 1:10 dilution of the commercial topical agent.

The sample bathed in DI water represented a neutral pH oral environment for the material. Different concentrations of APF solutions represented different acidic oral environment conditions. The test samples were stored in a 37°C room to simulate oral temperature. It has been mentioned in a previous study that 24 h of immersion would represent 1 year of exposure to APF solution for 4 min every day^13^. Hence, the immersion test was performed for 11 d to represent exposure of restoration materials to APF for 10-11 years.

Surface morphology of zirconia rods from all the groups were analyzed at specific time intervals (“t”) (t=24 h, 120 h, 192 h, 264 h). In addition, aliquots of immersion media from groups 2-4 were collected during the same time interval to verify the dissolution of zirconia particles release over prolonged exposure to acidic aqueous environment.

### Mass loss experiments

Mass loss and change in dimension were recorded to verify if dissolution of zirconia ions/particles could occur under the experimental conditions. Weight and dimensions (length and width) were recorded periodically for 11 d (264 h) for all specimens using a microbalance and caliper, respectively. Weight loss percentage was calculated using Equation 1. Where %∆W represents the percentage of weight loss; the initial weight of each specimen, before immersion, is denoted by W_o_; and the weight of immersed zirconia rods every 24 h is indicated by W_T_.


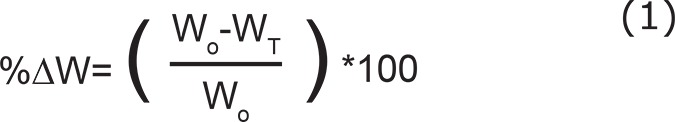


### Surface morphology

The effect of APF solutions on the material surface was analyzed using different microscopy techniques. Surfaces of all specimens were imaged using digital microscopy (Keyence VHX – 2000, Itasca, Illinois, USA), and analyzed by Scanning Electron Microscopy (SEM) (SEM, JEOL, JSM-6010, Peabody, Massachusetts, USA), before and after immersion.

### X-ray diffraction (XRD)

Zirconia samples were ground using an alumina mortar and pestle. Powder X-ray diffraction (Bruker D8 Advance Powder Diffractometer, Madison, Wisconsin, USA) was used to determine the structure of the material pre- and post- immersion. Diffraction data were collected for a 2-theta range from 10 to 80 at 0.1 step size on a powder diffractometer equipped with a LYNXEYE detector with a power of 40 kV and 40 mA.

### Zirconia release study

The release of zirconia particles due to surface degradation in the immersion solution was analyzed with an electrochemical biosensor chip platform that quantifies particle dissolution using Electrochemical Impedance Spectroscopy (EIS). Aliquots of testing solutions were collected before and after the immersion of zirconia rods. After the immersion of rods, sample aliquots were collected from the testing media at different time intervals “t” hours (h) (t=0, 24 h, 120 h, 192 h, 264 h).

The EIS test was performed at a constant peak-to-peak voltage of 10 mV and a frequency of 250 Hz. The impedance of the sample of interest varies according to the conductivity of it, which depends on the concentration of ceramic particles in the electrolyte (i.e., a decrease in impedance indicates a more conductive medium). Deionized (DI) water which was not subjected to any immersion was used as the control baseline. The ratio of change in impedance of the sample medium analyte from baseline was calculated to generate a percentage change in impedance (ΔZ). Percent change for impedance was determined using Equation (2). This allowed the data set to be averaged and the standard deviation to be determined over three runs on three separate EIS chips for the 0.123% APF solution. Only one EIS measurement (one run *per* chip) was taken for the 1.23% APF gel due to the high viscosity of the medium. Where %∆*Z* represents the percent change in impedance; *Z*_*c*_ and *Z*_*I*_ represent the impedance value of the immersion solution before and after immersion of specimens, respectively.


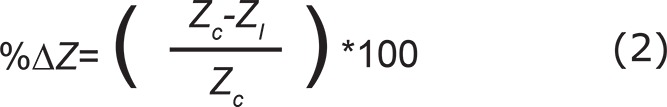


## RESULTS

### Mass loss analysis

Weight and dimension measurements of immersed zirconia samples showed only negligible change in both testing solutions. The average percentage of weight loss for samples immersed in 1.23% APF was about -0.01%, while samples immersed in 0.123% APF showed a weight loss of 0.01%. The dimensions of the specimens showed similar characteristics, where there was no change compared to pre-immersion values.

### Surface morphology

**Figure 1:**
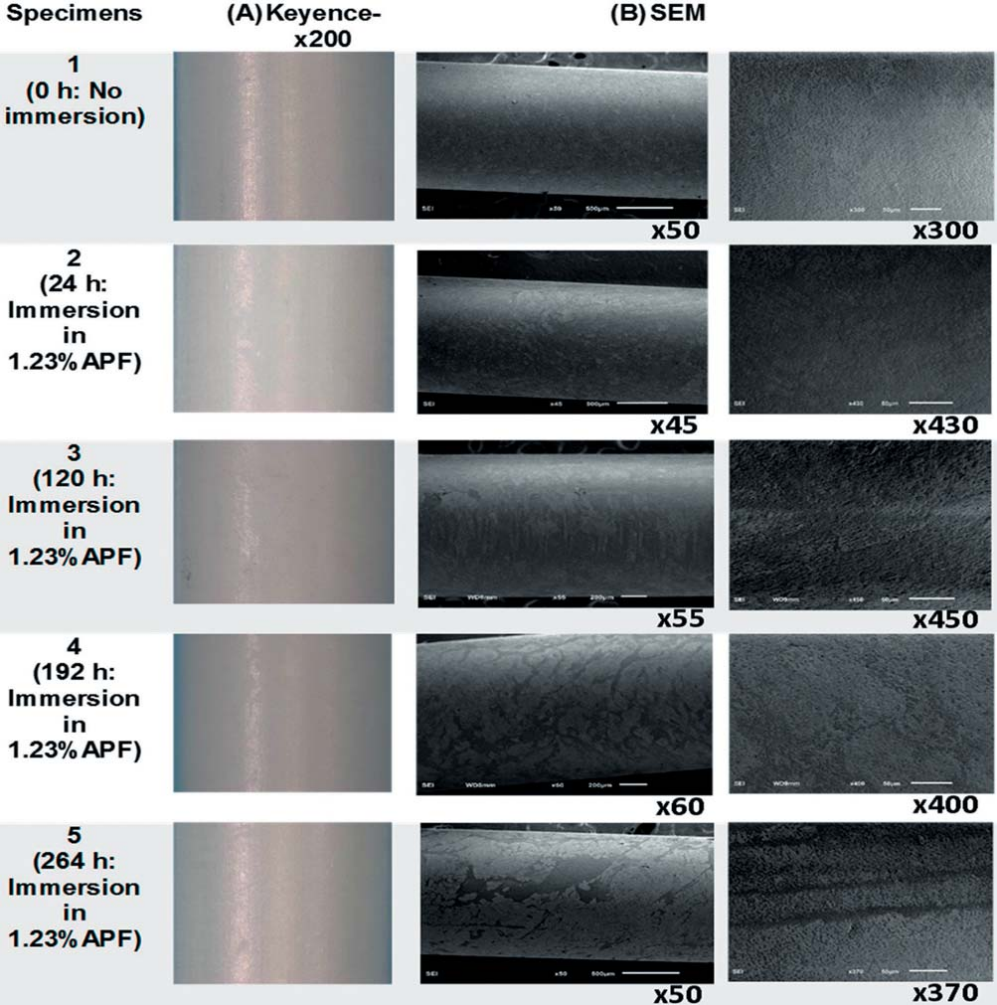
(A) Microscopic images at 200x magnification of zirconia surface before and after immersion in 1.23% Acidulated Phosphate Fluoride (APF) at 37°C for 24 h, 120 h, 192 h, and 264 h; (B) Scanning electron microscopy images of all zirconia specimens at different immersion times and solutions

**Figure 2:**
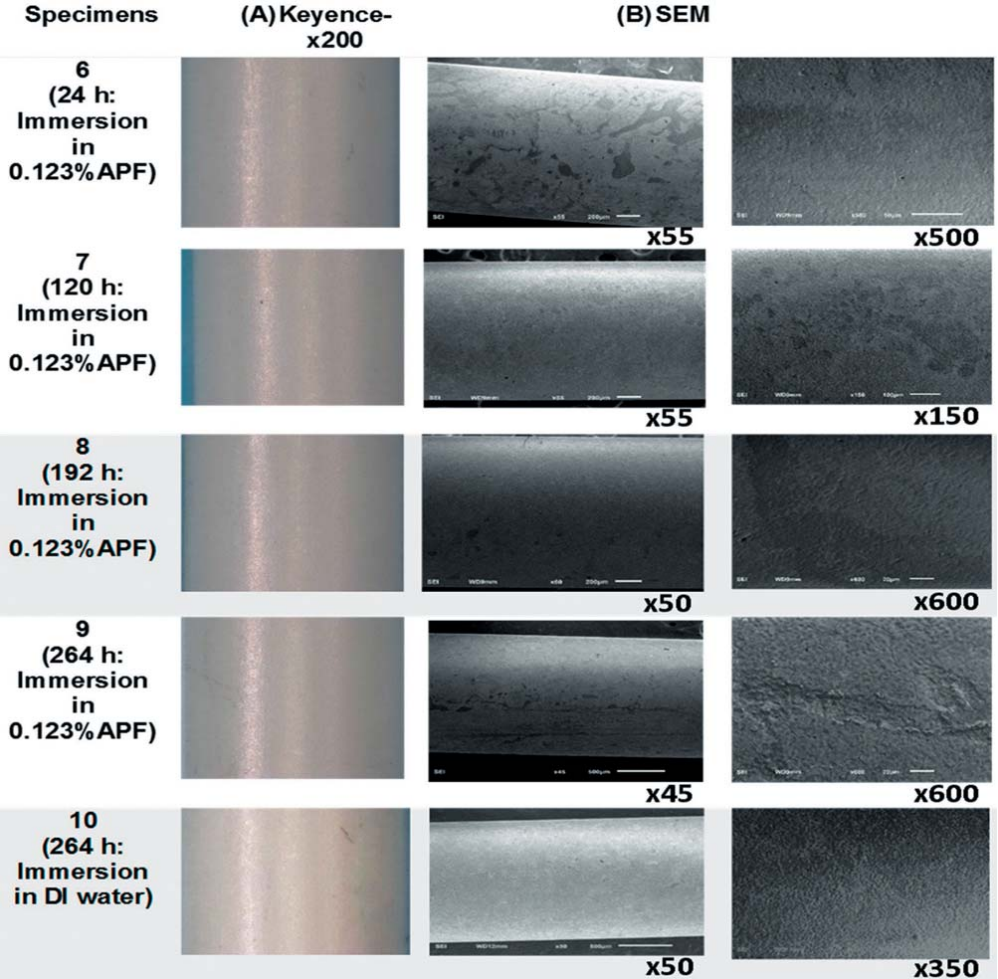
(A) Microscopic images at 200x magnification of zirconia surface after immersion in 0.123% Acidulated Phosphate Fluoride (APF) and deionized (DI) water at 37°C for 24 h, 120 h, 192 h, and 264 h; (B) Scanning electron microscopy images of all zirconia specimens at different immersion times and solutions

Digital microscopy images showed discoloration and loss of luster on the surface for the specimens immersed in APF solutions, as illustrated in Figure 1 and 2, when compared to the controls (Figure 1:1A and Figure 2:10A). Zirconia immersed in DI water showed similar luster to that of the control. Manufacturing marks were visible on all surfaces of the zirconia rods whether they were immersed or dry as seen in Figure 1:1A-5A and Figure 2:6A-10A.

SEM showed a smooth undisrupted surface for the control specimen (Figure 1:1B). The grain boundaries were visible and indicated a uniform top layer. With immersion in APF solutions, the specimens showed imperfections on the superficial layers. The disruption of surface grain and formation of micro pores increased with immersion time in both 1.23% and 0.123% APF solutions, as illustrated in Figure 1:2B-5B and Figure 2:6B-9B. Zirconia bathed in DI water was comparable to the control where an intact top layer was evident (Figure 2:10B).

### X-ray diffraction (XRD)

**Figure 3:**
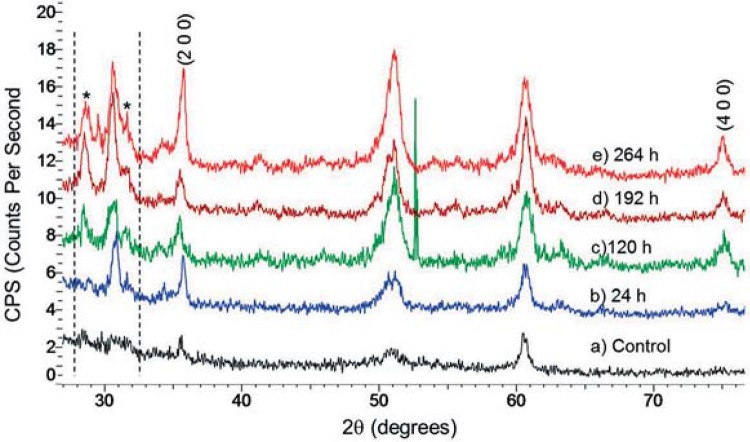
Powder diffraction patterns as a function of increasing immersion times in 1.23% Acidulated Phosphate Fluoride (APF) solution at oral temperature. (a) control (b) 24 h (c) 120 h (d) 192 h (e) 264 h, * indicates NaPO3

**Figure 4:**
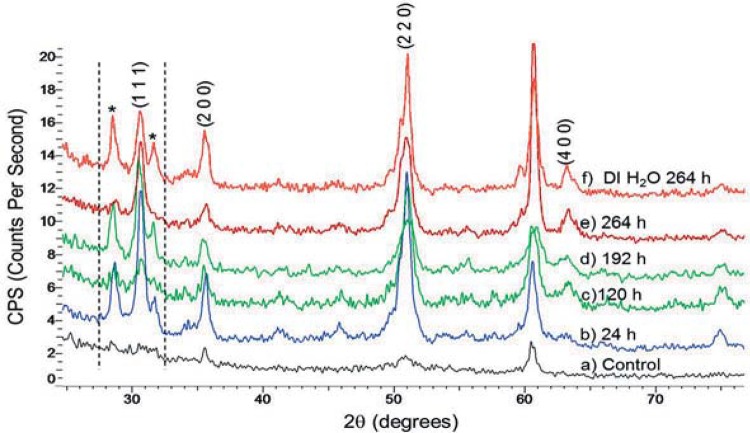
Powder diffraction patterns as a function of increasing soaking times in 0.123% Acidulated Phosphate Fluoride (APF) solution and deionized (DI) water at oral temperature. (a) control (b) 24 h (c) 120 h (d) 192 h (e) 264 h (f) DI water 264 h, * indicates NaPO3

XRD patterns for the control before immersion in DI water or APF solutions showed that zirconia was of the tetragonal phase as indicated in Figures 3a and 4a. This XRD pattern was used as a baseline for comparison to all the other specimens. For zirconia immersed in APF solutions, reflections corresponding to sodium phosphate (Na_3_PO_4_) are evident and indicated by an asterisk (*). As the immersion time increased, so did the intensities indicating the presence of higher concentration of sodium phosphate. Additionally, these peaks are more intense for the samples bathed in 0.123% APF solution (Figure 4) than in 1.23% APF solution (Figure 3). XRD patterns for the zirconia specimen in DI water showed peaks for sodium phosphate dehydrate (Na_2_HPO_4_.2H_2_O) (Figure 4f).

### Electrochemical Impedance Spectroscopy (EIS)-based biosensor

**Figure 5:**
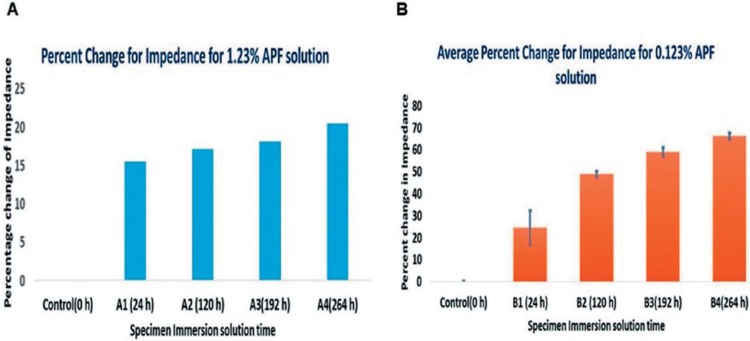
Electrochemical Impedance Spectroscopy- (A) Percent change in impedance for 1.23% Acidulated Phosphate Fluoride (APF) solution and (B) Average percent change for 0.123% APF solution, over 0 h, 24 h, 120 h, 192 h, and 264 h

Sample aliquots of immersion media such as DI water, 1.23% and 0.123% APF over the specified intervals of time (0 h, 24 h, 120 h, 172 h, 264 h) were analyzed using a biosensor chip platform. Impedance values obtained from the biosensor showed a decrease in impedance, with increasing immersion time of samples in APF, as shown in Figure 5A-B. This trend was consistent in the analysis of sample aliquots from 0.123% and 1.23% APF solution. This represents an increase in solution conductivity, which was evident with the increase in the percentage change in impedance %ΔZ. Its average increased by 20% in 1.23% APF and 65% in 0.123% APF media collected at t=264 h in comparison to the control solution before immersion. EIS measurements were acquired with three different chips for 0.123% APF solution and averaged (Figure 5B). Measurements were conducted only once for the 1.23% APF gel (Figure 5A).

## DISCUSSION

The immersion study of zirconia rods in APF was performed to investigate the effect of an acidic electrochemical environment on the surface of zirconia. It was hypothesized that acidic aqueous environment provided by the topical agent can degrade the zirconia surface and accelerate the process of ageing, leading to micro-crack nucleation and propagation.

Examination of surface morphology of samples exposed to APF containing media showed visible discoloration under digital microscope (Figures 1 and 2). This observation was attributed to a grain structural change in the superficial layer, which may have affected light reflection on the surface of the material. Manufacturing marks evident on all specimens were considered to be manufacturing defects, since they were visible on the non-immersed control as well as shown in Figure 1:1A-5A; Figure 2:6A-10A. Some of these deposits were etched out by APF from the specimens, leaving spots of black streaks (Figure 1:3A, Figure 2:6A and 9A. These superficial defects were evident in the SEM images, where the top layer seemed to be etched with apparent micro-pore formation (Figure 1:2B-5B; Figure 2:6B-9B). Similar disruption of the superficial layer and formation of micro-pores have been shown in studies where hot acidic surface treatment was applied on zirconia^5^. Previous literature studies suggest creation of microcracks in ceramic materials when exposed to ageing solutions^14^. This would further lead to several complications such as: (i) increase in surface roughness, which could increase plaque adhesion^22^, (ii) abrasion of antagonistic teeth^2^, and (iii) color change leading to improper aesthetic qualities of restorations^12^. APF at different concentrations has been reported to etch dental composites, porcelain, amalgam and dental cements *in vitro* ^7^ ^,^ ^9^. Ageing is characterized by the propagation of deformation from the surface to the bulk. It was essential to investigate the bulk crystal structure of zirconia samples to verify the extent of acidic attack.

XRD was performed to verify the crystal structure transformation. But, changes in lattice structure were not detected by XRD with the samples investigated. Further XRD analysis showed the absorption of sodium phosphate onto the surface of zirconia, but weight change was not evident as mentioned in the mass loss analysis. The disruption of the top layer and the absorption of sodium phosphate is a dynamic process in solution; therefore, change in mass was not measurable. The corrosion effect was limited to the superficial layers of the material and can be considered to be of nano-thickness. Samples immersed in 0.123% APF had a higher concentration of sodium phosphate with increasing time, as demonstrated by the increased intensities of Na_3_PO_4_ reflections in the XRD diffraction pattern (Figure 4A-E). This is because the diluted APF solution allowed the diffusion of ions to occur more freely unlike the 1.23% APF gel, whose high viscosity imposed a diffusion barrier for the movement of ions. Peaks of sodium phosphate hydrate seen for zirconia immersed in DI water (Figure 4F) were possibly a result of cross contamination of the APF immersion media caused by instruments used to extract the samples from the solutions.

Even though there was no change in the crystal lattice structure, both microscopic evidence and XRD results illustrated superficial surface degradation. Therefore, it was necessary to confirm the dissolution of zirconia particles in the immersion media. Sample aliquots of immersion media collected over specific interval of times were analyzed by an electrochemical biosensor chip platform. Electrochemical biosensor results showed more conductivity of the solution with increasing time, as illustrated in Figure 5(A-B). Correlations between EIS and XRD results showed the absorption of sodium phosphate onto zirconia for both concentrations of APF solutions. This phenomenon can be attributed to selective ion exchange of sodium phosphate from APF attaching to the surface of zirconia, leaving fluoride ions in the solution to allow for the formation of hydrofluoric acid (HF)^4^. Commercial APF (1.23%) has a fluoride content of 12,300 parts *per* million (ppm) with 0.34% of HF, where its dilution to 0.123% APF will provide a fluoride content of 1,230 ppm^8^ ^,^ ^30^. In addition, the ion exchange phenomenon leading to the formation of HF can further exacerbate the medium. HF present in the APF solution has been associated with the dissolution of silica in the dental bioglass ceramics. However, the lack of silica phase in zirconia has been attributed to its improved resistance to acid etching. But, a recent study suggested the possibility of etching of zirconia due to HF with an increased exposure time^24^. Earlier studies have disproved the theory of dissolution of zirconia or yttria into water and, similarly, showed no weight change in aged specimens^14^ ^,^ ^27^. Considering that our ageing solution was acidic, and that SEM images revealed disruption of a superficial layer, dissolution of this nano-layer is assumed to have occurred, which resulted in an increase in the conductivity of the APF solutions. This dissolution of ions was more prevalent for the 0.123% APF medium, which was attributed to the viscosity of the solution. The 1.23% APF gel was viscous, slowing the movement of ions and interaction between the gel and surface of material, while the diluted APF allowed for chemical exchanges between the surface and solution to occur more freely.

The results of this study clearly showed that the aqueous acidic environment was able to trigger superficial surface degradation with the dissolution of ceramic particles limited to the surface and did not affect the bulk crystal structure as confirmed by XRD, which was considered as an indicator for ageing (or) slow micro-crack propagation.

The degradatation limited to the surface without affecting the bulk crystal structure could explain the clinical finding of no zirconia framework fractures experienced in well conducted clinical studies, resulting in overall prosthetic survival rates of 100% up to 5 years^13^ ^,^ ^17^ ^,^ ^18^. Thus, the ongoing development of new clinical and laboratory procedures have yielded successful results of the PFZ as a viable restorative alternative to PFM, resulting in high patient satisfaction^19^.

However, superficial surface degradation should not be neglected. Due to the roughness induced by fluoride and acidic environments, the top layer could promote plaque retention creating a stage for peri-implant diseases to arise^22^. In Dentistry, zirconia is gaining importance as the material of choice for making abutments and crowns. Pozzi, et al.^19^ (2015), reported on zirconium oxide’s high biocompatibility and low plaque surface adhesion contributing to successful soft tissue integration, assessing a extremely low cumulative plaque score with 93% of patients without gingivitis throughout follow-up, and the remaining 2% and 5% with respective mild and moderate gingivitis^19^. Vice-versa, non-hygienic inaccessible restorations and roughed or not properly polished surfaces are significantly associated with implant loss and a high rate of peri-implantitis^22^.

It has also been suggested that formation of surface corrosion layers may influence the degree of degradation in addition to the effect of microstructure and environmental conditions^4^. Moreover, it has been reported that zirconia’s strength is influenced by different surface treatments that produce different degrees and types of surface damage^5^.

Certain limitations exist within this study. This methodology provides an overview on the corrosive behavior of APF on zirconia in oral temperature. Nevertheless, *in-vivo* oral environment can have fluctuations in pH, mastication forces, and other factors, that may further influence the materials’ properties and performance.

## CONCLUSION

This study investigated the effect of acidic aqueous electrochemical environment due to a topical solution. However, the oral environment is comprised of complex electrochemical factors such as saliva, enzymes, a wide variety of bacterial species, along with fluctuations in the pH and temperature conditions due to food particles and beverages. In addition, dental restoration materials *in vivo* are also exposed to mechanical factors such as occlusal forces due to mastication. It has been clearly stated that the mechanical properties of zirconia are affected under prolonged exposure in an aqueous medium. In the limited clinical evidences obtained so far, failure of zirconia has been primarily associated with fracture. Hence, future studies will include mechanical loading of zirconia in simulated oral environment to understand the interplay of mechanical and electrochemical factors that can accelerate degradation and fracture of the material.

Immersed samples did not show any transformation of crystalline phase from monoclinic to tetragonal. Hence, it was concluded that the acidic medium was not able to trigger the ageing phenomenon. However, microscopic images detected superficial surface degradation. In the oral environment, superficial surface damage, due to acidic medium, can be further invigorated by cyclic occlusal forces which can have a direct effect on the bulk properties of the material. Future studies will focus on the synergistic effect of mechanical forces and acidic electrochemical environment, which may accelerate crack propagation resulting in fracture and failure of the zirconia core or chipping of the porcelain veneering layer.
